# Opportunities and Problems of the Consensus Conferences in the *Care Robotics*

**DOI:** 10.3390/healthcare9121624

**Published:** 2021-11-24

**Authors:** Giovanni Maccioni, Selene Ruscitto, Rosario Alfio Gulino, Daniele Giansanti

**Affiliations:** 1 Centro Nazionale per le Tecnologie Innovative in Sanità Pubblica, Istituto Superiore di Sanità, 00161 Rome, Italy; giovanni.maccioni@iss.it; 2 Faculty of Engineering, Tor Vergata University, 00133 Rome, Italy; seleneruscitto@hotmail.com (S.R.); rosario.gulino.uni.tv@hotmail.com (R.A.G.)

**Keywords:** e-health, medical devices, m-health, rehabilitation, robotics, organization models, artificial intelligence, electronic surveys, social robots, collaborative robots, cyber risk, informatics, consensus conference, acceptance, clinical acceptance

## Abstract

Care robots represent an opportunity for the *health domain*. The use of these devices has important implications. They can be used in surgical operating rooms in important and delicate clinical interventions, in motion, in training-and-simulation, and cognitive and rehabilitation processes. They are involved in continuous processes of evolution in technology and clinical practice. Therefore, the introduction into routine clinical practice is difficult because this needs the stability and the standardization of processes. *The agreement tools*, in this case, are of primary importance for the clinical acceptance and introduction. The opinion focuses on the *Consensus Conference* tool and: (a) highlights its potential in the field; (b) explores the state of use; (c) detects the peculiarities and problems (d) expresses ideas on how improve its diffusion.

## 1. Introduction

The Policy Department for Economic, Scientific and Quality of Life Policies of the European Parliament identified the most interesting applications of the care robots (CR)s [1] divided into four groups:

- Robotic surgery
- Care and socially assistive robots
- Rehabilitation systems
- Training for health and care workers

### 1.1. The Care Robots: Advantages and Disadvantages

The discussion on the merits and demerits of robots is a current topic both in the world of industry and consumption [2,3] and in the *health domain* [4]. Employing robots in a *health domain* brings innumerable benefits and is equally advantageous for both healthcare providers and patients.

Robotic surgery, for example, has reduced the risk of infection, the blood loss, and improves the recovery time for the patients. The use of robotics in rehabilitation and assistance improves the care and decreases the professionals’ workload. The robots in the *health domain* near always are practical, useful, effective, and tireless. 

All this makes the use of robots particularly useful in the following applications [4]:

*Surgeries*—Robot-assisted surgeries are reliable, precise, flexible, and practical. They allow “minimally invasive” surgical actions.

*Clinical Training*—Clinical training robots are realistic simulation devices using also haptic systems very useful in the training.

*Prescription/Dispensing*—These robots can both dispense medicine at a very high speed and accuracy and similarly handle sensitive liquids or viscous materials.

*Care/Services*—These robots aid both to perform daily activities (for example moving, transport) and daily check-ups (like temperature, blood sugar, pressure).

*Disinfection and Sanitation*—These specialized robots carry important routine actions in the *health domain,* such as the air circulating and surface disinfection process.

*Telepresence*—They are telemedicine robots, designed to interact with the patient from remote locations. They can be also used in domotics.

*Logistic robots*—The logistics robots equipped with navigation systems perform basic tasks such as moving lunches or medications.

*Rehabilitation Robots and Nursing/Assistance Robots* are also other important robots generating significant interest. 

While there are innumerable benefits of employing robots to run tasks in the *health domain*, there are probabilities of faults. There is always some scope for human error or mechanical failure with these advanced robots. A single mechanical fault can cause physical damages/harms, psychological harm (for example the social robots) or even death. Another major disadvantage is the cost factor. The use of surgical robots or robots for rehabilitation and assistance is limited to the developed countries. Other problems are represented by the strong impact and implications of the ethics in this field [5,6].

### 1.2. The Strong Need of the Agreement Initiatives in Care Robotics

CRs are taking on an increasingly important role in the *health domain*. A simple search on Pubmed with key (*robot [title/abstract]*) [7] shows that to date (07/09/2021) 22,776 studies referring in some way to CRs are accessible in this Database. A total of 8693 (equal to 38.1%) of them have been published between January 2019 to date. There is no doubt that the CR sector is growing rapidly. The CR sector is a highly scientifically innovative sector subject to continuous research developments and innovations. 

Therefore, the standardization processes that lead to routine use in the clinical setting are in a state of constant chase. National and international regulations (governing development and certification processes and use in the clinical setting) are often of a general nature or not specifically designed for devices subject to continuous technological innovation.

Think, for example, the cybersecurity implications for these systems [8,9].

Furthermore, the organizational models for using CRs are different depending on the application. They differ not only from country to country, but also between regions and areas of the same country, or among application regimes (e.g., public, or private). This makes the results of scientific research (revisions for example) not immediately applicable or translatable, precisely because they may also depend on the organizational model where they have been applied. For example, Italy has a regionalized *health domain* organization, with different delivery methods, depending by region and on whether it is a public or private service provider [10].

Tackling the issue of acceptance (through proper tools is mandatory) into the clinical routine is a hot and current issue, with several implications of various fields, such as ethics, safety, cybersecurity, laws, and policy. It is a multidimensional problem in the *health domain* with several variables (e.g., the evolution of research, the regulatory framework, the organizational model, the acceptance and opinion of insiders, the cost-effectiveness, the training). 

An approach in this area certainly starts from evidence-based medicine (EBM) and then must use agreement tools.

The EBM [11,12,13] aims to guarantee all patients the same quality, efficiency, and effectiveness of intervention, overcoming some limitations of the individual experience of clinicians. In recent years, the progress achieved by research allows for the constant and updated production of new knowledge. Useful tools for disseminating knowledge have been developed such as *systematic reviews*, *meta-analyses*, *reviews of literature*, *decision-making systems based on formal models*. Since the 1980s, to respond even more precisely and punctually to these knowledge transfer needs and to produce useful recommendations for guiding clinical practice, “*guidelines*” (GL) [14], “*technology assessment*” (TA) reports [15,16,17] and “*consensus conferences* [18,19] are born. The *Consensus Conferences* (CC)s are tools, allowing, through a road map (based on a formally shared and structured process), an agreement on a topic. The purpose of the CC is to produce evidence-based recommendations, useful to assist operators and patients in a multidimensional domain. 

### 1.3. The Purpose and Structure of the Study

The goal of the *opinion* is to explore the use of the CCs on the CRs, identifying the state of application, the opportunities and-problems that have emerged, and providing reflections to scholars and stakeholders.

The study is organized into two sections plus the introduction (Section 1) and the conclusions (Section 4). Section 2 recalls, based on a brief review of the scientific literature, the relevance of the CC tool. Section 3 analyses (a) the state of application of this tool in Care Robotics. (b) The opportunities and-problems that have emerged. (c) The aspects that have been little or no dealt with. 

## 2. The Consensus Conferences: Brief Reminder and Relevance

The purpose of a CC is to produce evidence-based recommendations useful to assist operators and patients in a *multidimensional domain*. The CC agreement tool [18,19], can use, the output of both TA Reports (TR)s studies and GLs. However, it can also use other tools for monitoring or collecting opinions, such as *surveys* and *focus groups*. GLs and TRs are analysed by experts recruited for the CC. Compared to other tools, the CC allows, thanks to the possibility of breaking down the problem into specific questions, to share and precisely clarify the points on which there is greater uncertainty, thus laying the right premises to obtain targeted and precise answers, consult databases in a specific way, excluding that little or irrelevant part of the literature. The US National Institutes of Health established the Consensus Development Program in 1977 [20] with the aim of providing independent, impartial, and evidence-based assessments of complex medical issues, developing the consensus conference tool for the first time. The method, modified several times over the years, involves the interfacing of different actors and phases. In addition, other nations developed similar methods [20], as for example the France [21]. In Italy the Istituto Superiore di Sanità (The Italian NIH) produced a Manual for the CCs in 2008 [13] and followed several CCs as. 

### 2.1. An Organizing Committee Starts the Work of the Conference

One or more subjects interested in the chosen topic can play this role, including: institutions, scientific societies, experts in the sector in question, patient associations or their families. The a priori presence of a public institutional subject (for example in Italy the Istituto Superiore di Sanità, the Italian National Institute of Health) could benefit the entire process. The latter, as a neutral figure, would have the possibility of intervening in resolving any conflicting opinions of the interested parties in various capacities. Furthermore, this would favour the dissemination and application on the national territory of the produced recommendations. There are several actors in a CC. 

In addition to develop the conference, the *Organizing Committee* (OC) selects the members of both the *Technical-Scientific Committee* (TSC), the *Jury Panel* (JP), the experts of the *working groups* (WG)s and provide them with methodological support together with the TSC. 

### 2.2. The Working Groups (WG) Plays a Fundamental Role

The principal question, to be answered by the CC, will be divided into articulated *sub-questions*. Each *sub-question* will be assigned to a specific *W*G. 

These are composed of experts with very specific skills in relation to the subject examined. These are multidisciplinary groups, which, are required, (starting from the critical analysis and evaluation of the available scientific evidence), to summarize the latter and prepare reports with which to expose the data to the JP. In robotics, as in other fields, the success of a CC depends on how the *sub-questions* assigned to each *WG* are articulated.

## 3. The Consensus Conferences and the Care Robots: State of Application, Opportunities, and Problems

The sector of the CRs has experienced a significant and constant increase in recent years. However, there is a strong inhomogeneity in the criteria for clinical use, as well as in the evaluation of the outcomes. The CC tool could be greatly useful.

### 3.1. The State of Application

We investigated the application status of the CC. In line with this type of contribution, we did a e search on Pubmed with the key: (consensus conference) AND (robot [title/abstract])

The search [22] returns 17 results [23,24,25,26,27,28,29,30,31,32,33,34,35,36,37,38,39].

After an analysis of the records, 4 studies were discarded because they were not completely relevant. The 13 remaining references are the following [23,24,25,26,28,30,32,33,34,35,36,38,39].

Another search “with the key: consensus conference” shows [40] 21.779 results.

Specific search in the field of the application but without referring to robots returns:

- With the key: (consensus conference) AND (surgery) [Title/Abstract]) [41] 1.676 results.
- With the key: (consensus conference) AND (rehabilitation) [Title/Abstract]) [42] 372 results.
- With the key: (consensus conference) AND (assistance) [Title/Abstract]) [43] 94 results.
- With the key: (consensus conference) AND (training) [Title/Abstract]) [44] 800 results.

The results obtained in [22] is equal to a percentage ranging from 1.01% (in the case of surgery) up to 18.09% (in the case of assistance).

Table 1 highlights the detected sectors [23,24,25,26,28,30,32,33,34,35,36,38,39] dedicated to rehabilitation, diagnostics, therapy, and surgery. 

Appendix A.1, in Appendix A, reports the highlights from studies in Table 1 in details. 

### 3.2. Opportunities and Emerging Problems 

From a general point of view, it emerges (Table 1 and Appendix A.1) that the CC tool is currently used in the context of the CRs. CCs were used in all the applications in [1], except for Social Robots, probably because this type of CR is the most recent and is the one most subject to technological updating. The studies also highlight that the CCs have been an important opportunity for the connection between experts operating in various fields, from bioengineering up to clinics. Each CC, reported in the Table 1, Appendix A.1, faces the robotics in a highly specific clinical question; this allows experts to better focus on a question and investigate the problem in more detail. The CC deals with a specific topic, as for example [23] *the robot* in *neurological rehabilitation (a specific sector of rehabilitation)*. Again, for example, the *surgical robot* in *Radical Cystectomy (a specific surgical sector)* [32]. In some cases, the CR is not the direct focus of the CC. Nevertheless, its important role and its innovation is faced [26,30]. Among the methodological tools used in the CCs, we also find, not only the review, but also the surveys and the focus groups [26,32,33,34]. It also emerges that the CCs involved scientific societies locally, or regionally (e.g., Europe, Asia). In the articles in Table 1, however, the activities of the CCs are only partially reported. Furthermore, it is not possible to make a constructive comparison between the various studies. The contents relating to the total output of the CC are therefore not available on Pubmed. Probably because the final documents, when available, have been disseminated through different editorial tools (e.g., national monographs or by scientific societies or by sponsoring bodies or guarantor bodies). In consideration of the previous point, we should consider that the *medical knowledge* must be made also by accessing and monitoring the web of the scientific societies/associations (sponsoring or supporting the initiatives) or of the guarantor bodies. This makes the work of experts and researchers difficult to carry out with obvious difficulties due to both the fragmentation and the relative retrieval of documentation. The search on the Web, confirms this. It highlights how the information on the CCs is distributed and spread over various resources. It can be found, partially, dynamically updated, on the websites of the sponsoring scientific societies [45], on the website of the Guarantor Body [46], or on other national databases [47]. We have also extended a web search to technologies that can somehow intersect with CRs. We have noticed that Assistive Technologies can intersect with the CRs, starting from the definition [48,49]. Also, in this case, CCs are reported on the web [50,51]. Surely, these latter considerations, on the information availability [45,46,47,48,49,50,51], highlight how the attention must also be shifted outside of Pubmed, monitoring, and tracking the web of the scientific societies, associations and guarantor bodies.

### 3.3. Aspects Not Adequately Explored in the Consensus Conferences in Robotics

Based on what emerged from the research in the literature there are aspects that have not been adequately explored on the CC in this sector. Indeed, two further issues need to be deepened for the CRs (compared to other biomedical technologies).

1. *The implications with ethics, regulatory aspects and in the new emerging risks (for example Cybersecurity).Ethics* has an important role and a peculiarity on the CRs, such as on the SRs. The ethical issues on CRs have identified two *macro-sectors* [5,6]. The first *macro-sector* is the ethics in a responsible research and innovation [5]. The second *macro-sector* is the ethics problem encountered while building moral CRs [6]. There are *shadows* in EU Medical Device (MD) regulations [52]. *First*, they focus a lot on manufacturers and little on recipients/users. *Second*, the intended use and certification [53], must be aligned, and this is not always easily feasible in the field of the medical devices; for this reason the *health domain* supervisory systems are always active with monitoring actions. There are limits in the application of specific Cyb certifications. They are voluntary, as in the case of the Cybersecurity ACT [54]. The CRs would need an ad hoc regulatory framework, in consideration of the peculiarities [8].
2. *How to organize the WGs considering the peculiarity of the CR*. The organization of the *WG*s has a basic importance. The principal question, to be answered by the CC, is, indeed, divided into articulated sub-questions. In robotics, as in other fields, the success of a CC depends on how the *sub-questions* assigned to each *WG* are articulated.

The key queries that a CC must answer in a specific clinical application of specific CRs, also considering the previous point, (based on current scientific knowledge and experience gained in the recent years), are the following:

- Definitions and classification criteria for the devices based on the intended use.
- Indications on the specific clinical use of devices in clinical applications.
- Scientific References and consolidated experience for the development of the CRs.
- Organizational contexts and changes in the workflow.
- Regulatory framework (including the cybersecurity) and ethical issues for the devices.

The literature research did not provide always-clear suggestions on the WGs organization.

However, in [47] we can find a proposal of WGs for a specific CC in neurorehabilitation, which also considered some of the above listed issues. This is useful also to stimulate other CCs.

Based on this and the above considerations our *opinion* is that a set of *WGs* can be defined as in Appendix A.2 (Appendix A), for example, for a generic CC raging from robots for neurosurgery up to social robotics.

## 4. Conclusions

The sector of the CRs has experienced a significant and constant increase in recent years. However, there are strong inhomogeneities in the criteria for clinical use, as well as in the evaluation of their outcomes. The national and international bodies must contribute to concretize the efforts of national and international research. They must make available through consensus initiatives, their skills in the technical–scientific–regulatory field. The agreement tools are therefore strategic for this. The CCs are agreement tools, allowing, through a road map [18,19,20,21] (based on a formally shared and structured process), an agreement on a complex and articulated topic. They produce evidence-based recommendations, useful to assist operators and patients in a multidimensional domain. They have a strong potential on CRs because they can focus on a specific task (e.g., a specific clinical application of a specific CR), stress it, and considers a large set of implications. We first investigated the use of CCs in care robotics. 

The analysis of the scientific literature has highlighted some *lights* and *shadows*. 

*Among the lights,* we highlight the following: (a) the CC tool, [23,24,25,26,28,30,32,33,34,35,36,38,39], was used in the fields of *rehabilitation, diagnostics, therapy, and surgery*, on all applications of consolidated CRs [1], (with the exception of Social Robots, which are still subject to technological stabilization processes). (b) The CCs were the opportunity to: (1) bring together national and international experts from different cultural backgrounds in national and international meetings, (1) make them work around a structured and wide-ranging review process. This guarantees a broad consensus. (b)There has been a wide involvement of national and international scientific societies dealing with the application. (c) The methodologies to be used have been individuated. In addition to the consolidated reviews of the literature, the methodologies that can operate on the territory and on the groups involved were also indicated (for example questionnaires and focus groups).

Among the *shadows,* we highlight the following: (a) the studies available on the leading *health domain* databases (e.g., Pubmed) do not report the definitive documents but only partial considerations. Information is scattered on the networks, for example, partially available on the webs of the *scientific societies/associations* sponsoring the initiatives or of the guarantor bodies. This makes the work of experts and researchers difficult to carry. (b) The implications with ethics must be deepened. Ethics has an important role and a peculiarity on CRs, ranging from the ethical issues in research and innovation [5] up to ethics problems encountered while building moral CRs [6]. (c) The limits of the EU Medical Device (MD) regulations [52] must be considered, as: (1) the regulations focus a lot on manufacturers and little on recipients/users. (2) The intended use and certification [53], sometimes, are difficult to align. (d) The problems of the new emerging risk of cybersecurity must be carefully considered. The certification on the cybersecurity, according to the Cybersecurity ACT [54], is voluntary. Therefore, CCs must express clear suggestions for this. (e) It is not easy to find clear indications on how organizing *WGs* in a CC. The organization of the *WGs* has a basic importance. In robotics, as in other disciplines, the success of a CC depends on how the WGs are articulated. 

### Final Reflection

There is an increasing policy interest in renovating healthcare in a way comparable to how robotics transformed the industry, in terms of augmented productivity and resource effectiveness and productivity.

The introduction of these systems in clinical practice, and more generally in the *health domain*, requires great efforts of agreement through appropriate tools that consider all dimensions of the problems (e.g., clinical, regulatory and cost-effectiveness). In our study, we investigated the use, criticalities, and opportunities of the CCs.

International stakeholders need to undertake:

- Initiatives of greater diffusion of the methodology through tools that give it wide visibility.
- Census and categorization of the past and ongoing CCs.
- Coordination and initiation of CC programs at both national and international level involving the greatest number of experts.

## Figures and Tables

**Table 1:** Application of the consensus conference in robotics.

Reference	Application
[23,24,25]	Robotics in neurorehabilitation
[26]	Robots used in transcranial magnetic stimulation
[28]	Training in robot-assisted Surgery
[30]	Robotics on laparoscopic liver resection.
[32,33,34]	Robot assisted radical cystectomy
[35,36]	Robot assisted radical prostatectomy
[38]	Robotic pelvic surgery
[39]	Robots used in simulation environments

## Data Availability

Not applicable.
